# Cellulolytic potential under environmental changes in microbial communities from grassland litter

**DOI:** 10.3389/fmicb.2014.00639

**Published:** 2014-11-25

**Authors:** Renaud Berlemont, Steven D. Allison, Claudia Weihe, Ying Lu, Eoin L. Brodie, Jennifer B. H. Martiny, Adam C. Martiny

**Affiliations:** ^1^Department of Earth System Science, University of California, IrvineIrvine, CA, USA; ^2^Department of Biological Science, California State UniversityLong Beach, CA, USA; ^3^Department of Ecology and Evolutionary Biology, University of California, IrvineIrvine, CA, USA; ^4^Ecology Department, Earth Sciences Division, Lawrence Berkeley National LaboratoryBerkeley, CA, USA; ^5^Department of Environmental Science, Policy and Management, University of CaliforniaBerkeley, CA, USA

**Keywords:** cellulase, metagenomics, leaf litter, global change, microbial community composition

## Abstract

In many ecosystems, global changes are likely to profoundly affect microorganisms. In Southern California, changes in precipitation and nitrogen deposition may influence the composition and functional potential of microbial communities and their resulting ability to degrade plant material. To test whether such environmental changes impact the distribution of functional groups involved in leaf litter degradation, we determined how the genomic diversity of microbial communities in a semi-arid grassland ecosystem changed under reduced precipitation or increased N deposition. We monitored communities seasonally over a period of 2 years to place environmental change responses into the context of natural variation. Fungal and bacterial communities displayed strong seasonal patterns, Fungi being mostly detected during the dry season whereas Bacteria were common during wet periods. Most putative cellulose degraders were associated with 33 bacterial genera and predicted to constitute 18% of the microbial community. Precipitation reduction reduced bacterial abundance and cellulolytic potential whereas nitrogen addition did not affect the cellulolytic potential of the microbial community. Finally, we detected a strong correlation between the frequencies of genera of putative cellulose degraders and cellulase genes. Thus, microbial taxonomic composition was predictive of cellulolytic potential. This work provides a framework for how environmental changes affect microorganisms responsible for plant litter deconstruction.

## INTRODUCTION

Establishing the connection between community structure and function has been a longstanding yet elusive goal in microbial ecology. Making such a connection is especially critical for predicting how communities and functions will respond to global environmental change (Allison and Martiny, 2008; Trivedi et al., 2013). Meeting this challenge depends on linking traits that control responses of microbial taxa to the environment (“response” traits) with those that determine ecosystem function (“effect” traits; Lavorel et al., 1997; Gross et al., 2009; Webb et al., 2010). This linkage has been elusive due to the difficulty of isolating microorganisms and characterizing their traits in complex communities. The advent of high-throughput – omics approaches offers a means of linking response and effect traits in microbial communities. When coupled with experimental manipulations of environmental conditions, (meta)genomic approaches can provide a window into both microbial community responses and concurrent changes in functional potential.

Local communities are increasingly being confronted by global-scale environmental changes. For instance, drought and nitrogen deposition are predicted to affect many ecosystems (Cook et al., 2004; Hole and Engardt, 2008; Fenn et al., 2010; Seager and Vecchi, 2010). Nitrogen deposition is known to change plant diversity and to increase primary production (Lamarque et al., 2005; Cleland and Harpole, 2010), whereas drought can lead to reduced primary production and plant diversity (Mueller et al., 2005). Previous studies have shown that microorganisms also respond to changes in water availability (Pesaro et al., 2004; Sheik et al., 2011; Cregger et al., 2012; Barnard et al., 2013; Bouskill et al., 2013). Similarly, N-addition can promote the growth of copiotrophic microbes, whereas other lineages may be negatively affected (Fierer et al., 2012; Philippot et al., 2013). However, we currently have a limited understanding of how environmental changes directly impact the genomic diversity of microbial communities and their associated functional potential.

Plant polymer degradation is a key microbial function that channels plant litter into microbial biomass, where it can be mineralized to CO_2_ or stabilized as soil carbon (Cebrian, 1999). Cellulose is one of the most abundant polymers in plant litter, and therefore the breakdown of this compound is a key step in the decomposition of plant material. Cellulose-degrading microbes produce cellulase enzymes that catalyze the first step of cellulose hydrolysis and release oligosaccharides that are accessible for many other lineages (Lynd et al., 2002; Goldfarb et al., 2011; Berlemont and Martiny, 2013).

Cellulases belong to glycoside hydrolases (GH) families 5, 6, 7, 8, 9, 12, 44, 45, and 48 (Berlemont and Martiny, 2013). These enzymes are frequently associated with carbohydrate binding modules (CBMs) assumed to increase cellulose hydrolysis. In both isolated microorganisms and complex microbial communities, the redundancy of seemingly similar proteins is assumed to promote synergistic interactions among enzymes with varying regulatory mechanisms and/or biochemical properties (substrate specificity, pH, etc.; Wilson, 2011).

Bacteria that carry cellulase genes are commonly associated with specific genera within the phyla *Acidobacteria*, *Actinobacteria*, *Proteobacteria*, *Bacteroidetes*, and *Firmicutes* (Haichar et al., 2007; Ulrich et al., 2008; Schellenberger et al., 2010; Goldfarb et al., 2011; Barnard et al., 2013; Berlemont and Martiny, 2013). The initial enzymatic breakdown of cellulose typically results in the release of oligosaccharides like cellobiose. To use oligosaccharides, microorganisms need to express β-glucosidase, which is associated with GH families 1 and 3. A recent genomics analysis suggests that more than 80% of sequenced bacterial lineages carry β-glucosidase (Berlemont and Martiny, 2013) and therefore most lineages may opportunistically benefit from the enzyme production of cellulose degraders.

In arid and semi-arid ecosystems, cellulose degradation and litter decomposition rates may depend on the responses of cellulose-degrading lineages to water and nutrient availability. Previously at our study site in a semi-arid California grassland ecosystem, experimentally induced drought significantly reduced litter decomposition rates and bacterial biomass (Allison et al., 2013). Likewise, decomposition rates and bacterial biomass were markedly lower during the summer dry season. Nitrogen addition had weaker effects on litter decomposition, but there was some evidence for adaptation of microbial communities to nitrogen availability. These results raise the question of whether changes in the abundance of cellulose-degrading lineages contributed to changes in overall litter decomposition rates.

To understand how cellulose degradation might respond to environmental changes, we identified the microbial metagenomic content in leaf litter across seasons and under experimentally manipulated water and nitrogen availability. Specifically, we aimed to characterize the genetic diversity of the leaf litter microbial community, the organisms carrying cellulases, and cellulase genes. We hypothesized that most of the cellulolytic potential (i.e., the collection of detected genes coding for cellulases) would be associated with fungal lineages, consistent with past studies of leaf litter (Schneider et al., 2012). Next, we investigated how cellulolytic potential responded to seasonal and experimental precipitation reduction in this environment. We hypothesized that cellulolytic traits would be correlated with responses to precipitation and nitrogen availability owing to physiological tradeoffs. Enzyme expression requires cellular resources, particularly nitrogen, so cellulolytic traits should be more prevalent in nitrophilic, copiotrophic taxa (Treseder et al., 2011). In contrast, cellulolytic traits should correlate negatively with drought tolerance due to the high resource cost associated with cell walls, osmolytes, and other tolerance traits. Finally, we evaluated if the environmental responses of microbial lineages were correlated with changes in cellulolytic potential. We hypothesized that the frequency of cellulolytic traits in the microbial community is predictable based on the taxa present in the community (e.g., at the genus level). Such a relationship is expected if cellulolytic traits are phylogenetically conserved and would be useful for linking cellulose degradation with other traits conserved among microbial taxa (Allison and Martiny, 2008; Berlemont and Martiny, 2013; Martiny et al., 2013; Zimmerman et al., 2013).

## MATERIALS AND METHODS

### FIELD EXPERIMENT

**FIGURE 1:**
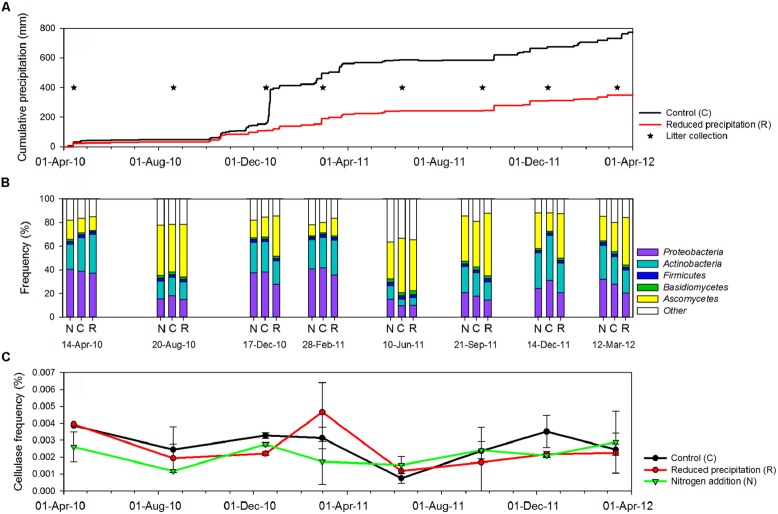
**(A)** Cumulative precipitation at Loma Ridge study site. **(B)** Overall distribution of sequences with annotation in the major microbial phyla detected in the leaf litter metagenomes across treatments [control (C), added nitrogen (N), reduced precipitation (R)] and seasons. **(C)** Fluctuations of the overall frequency of cellulase sequences in the leaf litter metagenomes.

Analyzed plant litter was collected at the Loma Ridge experimental field in Southern CA, USA (33°44′N, 117°42′E, 365 m elevation; Allison et al., 2013). Plant composition was dominated by exotic annual grasses (e.g., *Avena*, *Bromus*, and *Lolium*) and forbs (e.g., *Erodium*). The climate is semi-arid (mean annual precipitation of 325 mm) with most of the precipitation occurring between October and April (**Figure 1A**). Treatments were ∼50% reduction in precipitation (R), nitrogen addition (N), and control (C). Precipitation and nitrogen manipulations have been established there since February 2007. Precipitation reduction was achieved by covering plots with clear polyethylene during the rain events each winter. Nitrogen was added as 20 kg N/ha (CaNO_3_) prior to the growing season (October to April) and 40 kg N/ha (CaNO_3_) ∼3 months after the start of the growing season. Changes in plant community composition, litter chemistry, and microbial biomass across seasons and treatments were previously reported (Allison et al., 2013). For sequencing, 16 samples of plant litter from haphazardly located 0.07-m2 quadrats in each treatment (R, N, and control) were collected, once per season.

### DNA SEQUENCING AND TAXONOMIC ANNOTATION

To balance replication with the cost limitations of sequencing, we pooled eight plots from each treatment (each plot sampled and extracted separately) into two replicate samples for sequencing. This approach allowed us to capture spatial variation across our study site, while still keeping two replicates per treatment. Although two replicates is not ideal, we also sampled those pooled replicates eight times over the course of the experiment (i.e., April 14th, August 20th, and December 17th 2010; February 28th, June 10th, September 21th, and December 14th 2011; and March 12th 2012), which provided us with additional statistical power to capture treatment and temporal effects (see Results).

For each sampling date, eight leaf litter samples (∼20 gr) were collected, grinded in a mixer, subjected to direct DNA isolation as described before (DeAngelis et al., 2010), and normalized to the amount of leaf litter material used for extraction. After fragmentation to 300 bp using Covaris, equal amounts of DNA extracts were pooled to two replicates and prepared for sequencing. In total, 216 litter samples were processed and 54 metagenomic libraries [3 treatments × 8 dates × 2 replicates = 48 samples, two sequencing controls, and four samples from the dual factorial treatment (RN)] were prepared using a Truseq library kit (Illumina, San Diego, CA, USA) and sequenced with an Illumina HiSeq2000 (100 bp-paired ends). Sequences were treated as single reads for downstream analysis. Sequences were uploaded onto the MG-RAST server and made publically accessible (Table S5; Glass and Meyer, 2011). Finally, 107.4 Gbp (passed QC) were obtained.

The taxonomic (and functional) diversity of complex plant litter microbial assemblages was investigated using annotations of all the reads (i.e., WGS) in order to evaluate and compare samples without potential assembly biases based on composition or coverage. Taxonomic annotation, using the M5NR database, at the genus level, was considered for sequences with e-value ≤10^-5^. Using this cut-off, 53% of the sequences were annotated (Figure S1). After rarefaction, the distribution of taxonomically identified reads, was used to estimate and compare the taxonomic composition of samples.

### GLYCOSIDE HYDROLASES/CARBOHYDRATE BINDING MODULE IDENTIFICATION

In order to identify all the sequences associated with GHs and carbohydrate binding modules (CBMs) in the samples, sequences for each GH/CBM family, as defined in the CAZy database (Lombard et al., 2014), were extracted from the Pfam server and mapped against all sequenced genomes using SEED annotations (Overbeek et al., 2005; Berlemont and Martiny, 2013). The SEED functional annotation of these traits was then used as a reference to investigate the SEED-annotated sequences provided by MG-RAST output files (i.e., XXX_650.Superblat.expand.protein) for functional annotations. The resulting hits and their corresponding sequences were then subjected to a Pfam_scan analysis (PfamA db, e-value < 1e-5; Finn et al., 2014) to confirm functional annotations. This approach allowed us to identify short sequences from metagenomes matching GH/CBM from sequenced bacterial genomes.

### STATISTICAL ANALYSIS

Statistical analyses were performed using ‘Stat,’ ‘LME,’ and ‘Vegan’ packages in the R software environment (Pinheiro and Bates, 2000; Oksanen et al., 2012; R Development Core Team, 2012). We focused on describing the effect of date and treatment on groups defined taxonomically (phylum and genus level) and functionally (reads for cellulases). First the sequences were rarefied (*n* = 1,265,787 reads) and pairs of analytical replicates (from February 2011) were averaged. Next, sequences were binned by phylum/genus based on the taxonomy of the best hit in the M5NR database within the MG-RAST environment (e-value < 1e-5; Wilke et al., 2012). Genera containing cellulase sequences were defined as potential cellulolytic lineages. The genus-specific cellulolytic potential was defined as the total number of cellulase sequences from a genus divided by the total number of sequences from that genus across all samples.

Dependent variables, including taxon, and cellulase frequencies, were analyzed using analysis of variance on linear mixed effects models with repeated measures (Pinheiro and Bates, 2000). Fixed effects in the model included treatment (control, added N, or reduced precipitation), date, and the treatment by date interaction. The models included the combination of treatment and pooled replicate as a random effect with six levels.

The significance and magnitude of precipitation effects on the frequency of individual taxonomic groups, functional groups (e.g., potential cellulose degraders), and functional traits (e.g., cellulases) were investigated using the Pearson correlation test. The effect of each experimental treatment on the frequency of taxonomic and functional groups was investigated using a paired Welch-*t*-test to compare treatment means with control means.

For each bacterial genus, we determined the response to nitrogen addition and reduced precipitation. The response to treatment was defined as the ratio of the genus frequency in the treatment versus the control. We used the correlation coefficient (Spearman) between the log of the response ratio and the rarefied cellulase abundance for all genera to test for a link between treatment responses and cellulolytic potential.

## RESULTS

### MICROBIAL DIVERSITY

We identified microbial diversity using the taxonomic annotation of all the sequences. Sequences affiliated with Fungi accounted for 9–55% of the annotated reads, depending on the sampling season (**Figures 1A,B**). Sequences from *Ascomycetes* and *Basidiomycetes* dominated Fungi in all samples and accounted for 92.8 and 7.0% of the fungal hits, respectively. Sequences were affiliated with 422 detected families of Fungi. The most abundant Ascomycetes belonged to the families *Pleosporaceae*, *Phaeosphaeriaceae*, *Trichocomaceae*, *Nectriaceae*, and *Sordariiaceae* and accounted for 26.5, 19.8, 14.7, 4.9, and 4.6% of the fungal sequences, respectively. *Basidiomycetes* were primarily composed of the families *Tremellaceae*, *Ustilaginaceae,* and *Tricholomataceae* (2.3, 1.0, and 0.8% of the fungal sequences). 0.5% of the identified sequences were affiliated with *Archaea*, mostly *Euryarchaeota*. Bacteria accounted for 21–88% of the taxonomically identified sequences. *Proteobacteria*, *Actinobacteria*, and *Firmicutes* dominated the bacterial community and represented 43.9, 36.3, and 5.0% of the sequences for bacteria, respectively.

### CELLULOLYTIC TRAIT DIVERSITY

We next identified the distribution of GHs and carbohydrate binding module sequences (CBMs). Using a custom bioinformatics analysis, we detected 442,457, and 17,922 sequences for potential GHs and CBMs, respectively (Figures S1A,B; Tables S1 and S2). Across all samples, GHs accounted for 0.093% of the annotated sequences. Assuming at least few thousands genes per genome, this frequency of GHs indicated that many (if not most) microorganisms contained enzymes from this super-family. Most of the detected GHs appeared to be novel and had low similarity to known enzyme sequences (Figure S1C). Among the identified sequences for putative GHs, 92.7, 0.5, and 6.1% were likely to be derived from *Bacteria*, *Archaea*, and *Eukaryotes*, respectively. For Eukaryotes, 4.3 and 1.5% were likely affiliated to *Ascomycetes* and plants (e.g., *Brassica*), respectively. Sequences for enzymes involved in the processing of oligosaccharides were abundant (e.g., α- and β-glucosidases from GH1-3), whereas enzymes targeting complex structural polymers were less common (Figure S1A). For example, cellulases constituted 0.0025% of the annotated sequences, and thus, only a subset of the microbial organisms appeared to carry this trait. GH6 and 9 were the most abundant cellulase families, followed by GH8, 5, 12, 44, 45, 48, and 7 (Figure S1A; Table S1).

**FIGURE 2:**
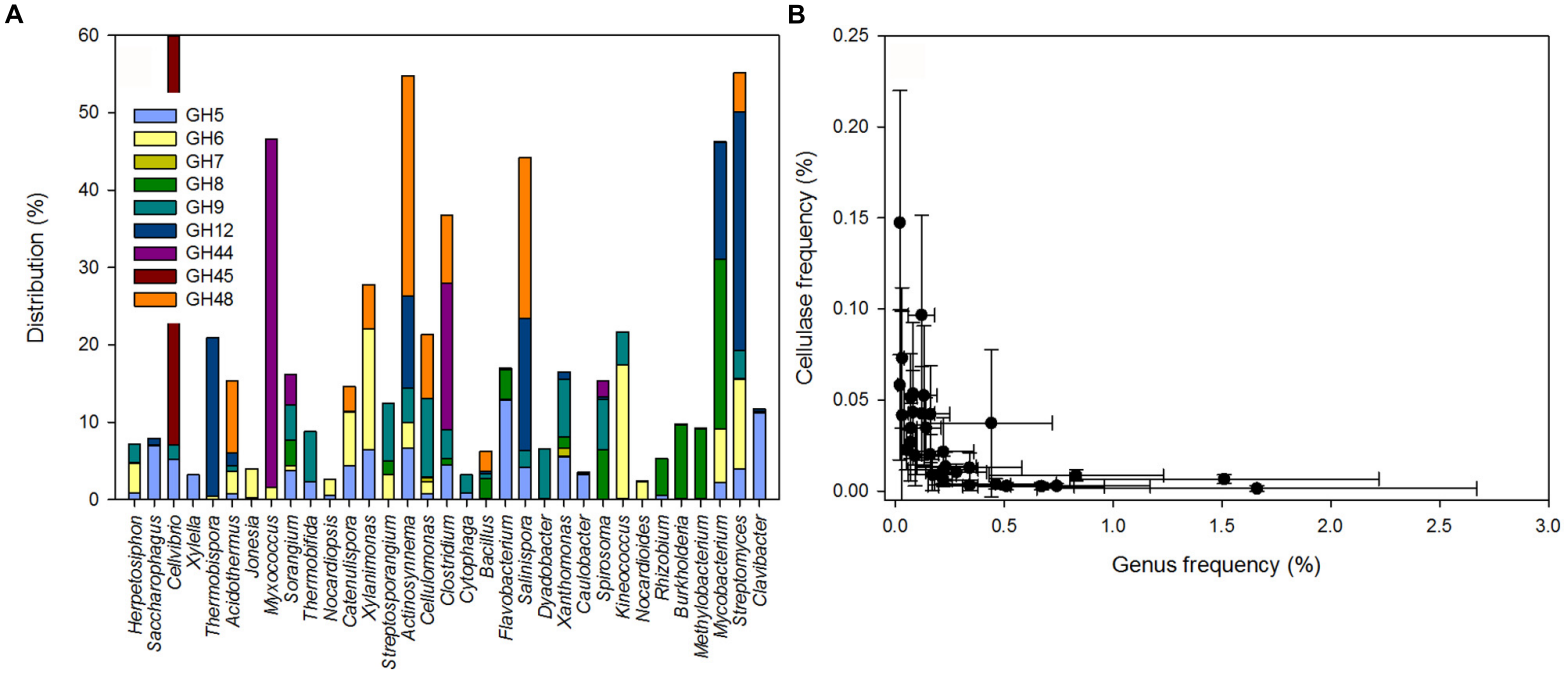
**(A)** Distribution of cellulolytic enzymes in potential bacterial cellulose degraders. **(B)** Relationship between the lineage specific cellulase frequency and the potential cellulose degrading genera in the leaf litter metagenomes (*P*_Spearman_ < 0.01).

A detailed analysis of the GH sequences involved in cellulose degradation revealed a vast diversity of enzymes likely to be derived from Bacteria and only few enzyme types from Fungi or plants (e.g., Brassica). Putative cellulase sequences were derived from lineages referred to as the potential cellulose degraders. Only 3.9% of the cellulases – mostly GH7 and to a lesser degree GH6 – were affiliated with the genus *Gibberella* (phylum *Ascomycetes*) and 3.7% (all within GH9) were derived from plants. In Bacteria, potential cellulose degraders included lineages within the *Actinobacteria*, *Proteobacteria*, *Firmicutes*, *Bacteroidetes*, and *Chloroflexi* phyla. 33 genera accounted for ∼88% of the reads for potential cellulases detected (**Figure 2A**; Table S3). Together, potential cellulose degraders were estimated to account for 18.2% of the taxonomically annotated sequences.

Some lineages were associated with only one GH family; for example *Clavibacter*, *Caulobacter*, *Xylella,* and *Saccharophagus* carried almost exclusively cellulases from GH5 (**Figure 2A**). Cellulases from *Methylobacterium*, *Rhizobium,* and *Burkholderia* were associated with GH8, and cellulases from *Nocardioides* and *Jonesia* were mostly associated with GH6. *Thermobispora* possessed enzymes associated with GH12 and a few with GH6. In contrast, other genera (e.g., *Streptomyces*, *Mycobacterium*, *Clostridium*, or *Sorangium*) carried cellulolytic enzymes from an array of GH families. Notably, some rare lineages had high numbers of cellulase genes. Across all samples, the frequency of reads from the bacterial potential cellulose degraders was negatively correlated with the corresponding genus-specific cellulase frequency (*P*_Spearman_ < 0.01, *r*_Spearman_ = -0.83; **Figure 2B**). Thus, our results showed that most of the reads for potential cellulase were likely to be derived from a limited set of bacterial genera. Some of these potential cellulose degraders were among the most abundant groups of bacteria detected (e.g., *Clavibacter* and *Streptomyces*) but some less abundant genera harbored a higher number of potential cellulases.

### MICROBIAL COMMUNITY FLUCTUATIONS

Monitoring communities seasonally over a period of 2 years allowed us to place environmental change responses into the context of natural climate variation. In order to investigate microbial responses, we ran a linear mixed effects model followed by ANOVA on the frequencies of sequences from taxonomically or functionally identified groups in each dataset. At the phylum level, the relative abundance of microbial lineages in the leaf litter metagenomes was significantly affected (*P* < 0.05) by the sampling date (Table S4). A few phyla (e.g., *Proteobacteria* and *Ascomycota*) were also marginally affected by the treatments (*P* < 0.1). Thus, our results suggested that, at the phylum level, the taxonomic diversity in leaf litter metagenomes was mostly affected by seasonally varying environmental factors and to a lesser extent by the experimental manipulations.

### SEASONAL PATTERNS

We investigated the effect of sampling date on overall microbial community composition and observed that the frequencies of sequences from Bacteria and Fungi displayed a seasonal pattern (**Figure 1B**). In the control, sequences from Fungi decreased from 44.2 ± 8.2% during the dry season (i.e., samples 20-August-10, 10-June-11, and 21-September-11) to 16.4 ± 6.4% during the wet season (*P*_Welch-t_ < 0.05). Sequences affiliated with Bacteria, including potential cellulose degraders and non-degraders, displayed the opposite trend, increasing from 36.1 ± 13.6 during the dry season to 70.6 ± 16.0% during the wet season (*P*_Welch-t_ < 0.05). The composition displayed similar temporal trends under reduced precipitation and added nitrogen treatments (**Figure 1B**).

### RESPONSE TO CHANGES IN WATER AND NITROGEN AVAILABILITY

Water availability was affected by seasonal and experimental changes (**Figure 1A**). In the control plots, with few exceptions (e.g., *Clavibacter*, *Kineococcus*, *Bacillus*, and *Clostridium*), the frequencies of the most abundant bacterial lineages were correlated with cumulative precipitation during the 2 weeks prior to litter collection. The frequencies of sequences from *Bacteroidetes* (*P*_Pearson_ < 0.05, *r*_Pearson_ = 0.89), *Proteobacteria* (*P*_Pearson_ < 0.05, *r*_Pearson_ = 0.75), and *Cyanobacteria* (*P*_Pearson_ < 0.05, *r*_Pearson_ = 0.76) were strongly positively correlated with precipitation, whereas *Actinobacteria* were weakly correlated (*P*_Pearson_ < 0.05, *r*_Pearson_ = 0.32). As a whole, potential bacterial cellulose degraders were significantly affected by precipitation (*P*_Pearson_ < 0.05, *r*_Pearson_ = 0.57). Fungal phyla displayed the opposite trends (*P*_Pearson_ < 0.05, *r*_Pearson_ = -0.72), and *Archaea* remained unaffected by the precipitation (**Table 1**; Table S3). This suggested that, among seasonally fluctuating parameters, precipitation strongly affected the microbial community in the leaf litter.

**Table 1:** Average frequency (%) of rarefied reads for the most abundant microbial lineages detected in leaf litter metagenomes.

	Control		Reduced precipitation		Nitrogen addition	
	Frequency (%)	Precipitation r_P_ (*P_P_)	Frequency (%; *P_Welch-_*_t_*)	Precipitation *r*_P_ (*P_P_)	Frequency (%; *P_Welch-_*_t_*)	Precipitation *r*_P_ (*P_P_)
**Sum of bacteria**	**61.46 ± 22.6**	0.67*	50.38 ± 20.58*	0.76*	**63.63 ± 18.14**	0.68*
*Proteobacteria*	27.96 ± 11.61	0.75*	21.81 ± 9.96*	0.81*	29.15 ± 10.46	0.74*
*Actinobacteria*	22.46 ± 9.67		19.5 ± 9.28		22.85 ± 6.41	
*Bacteroidetes*	5.29 ± 3.15	0.89*	4 ± 2.31	0.72*	6.15 ± 3.38	0.81*
*Firmicutes*	2.5 ± 0.19		2.4 ± 0.48		2.43 ± 0.25	
*Cyanobacteria*	0.76 ± 0.12	0.79*	0.64 ± 0.07*	0.69*	0.72 ± 0.12	0.61*
*Chloroflexi*	0.36 ± 0.09	0.55*	0.3 ± 0.06*	0.78*	0.33 ± 0.06	0.51*
*Acidobacteria*	0.36 ± 0.14	0.78*	0.27 ± 0.1*	0.70*	0.33 ± 0.11	0.69*
*Planctomycetes*	0.31 ± 0.09	0.64*	0.23 ± 0.05*	0.74*	0.28 ± 0.06*	0.56*
*Verrucomicrobia*	0.3 ± 0.1	0.72*	0.23 ± 0.06*	0.68*	0.27 ± 0.08*	0.60*

**Sum of potential cellulase degraders**	**19.36 ± 7.59**	0.57*	15.70 ± 6.76*		**19.42 ± 5.37**	0.60*

**Sum of Archaea**	**0.5 ± 0.05**		**0.45 ± 0.05** *		0.46 ± 0.06*	
*Euryarchaeota*	0.39 ± 0.03		0.34 ± 0.03		0.35 ± 0.04	
*Crenarchaeota*	0.1 ± 0.02		0.09 ± 0.02*		0.09 ± 0.02^+^	
*Thaumarchaeota*	0.01 ± 0		0.01 ± 0		0.01 ± 0^+^	

**Sum of Fungi**	**27.33 ± 15.12**	-0.72*	38.45 ± 15.52*	- 0.79*	**26.45 ± 12.65**	-0.72*
*Ascomycota*	25.39 ± 14.56	-0.72*	36.39 ± 14.95*	-0.79*	24.74 ± 12.13	-0.72*
*Basidiomycota*	1.89 ± 0.61	-0.56*	2.01 ± 0.73	-0.58*	1.67 ± 0.76	-0.54*
*Unclassified Fungi*	0.03 ± 0.02	-0.55*	0.03 ± 0.02	-0.70*	0.02 ± 0.01	-0.51*
*Glomeromycota*	0.01 ± 0	-0.68*	0.01 ± 0		0.01 ± 0	-0.55*

Significant treatment effects relative to controls are denoted with an asterisk (*P< 0.05, paired Welch *t*-test). Effect of cumulative precipitation (Precip., *r*_Pearson_) on the frequency of lineages in each treatment (*P_Pearson_ < 0.05).

Experimental precipitation reduction reduced the overall bacterial frequencies by ∼10% (*P*_Welch-t_ < 0.05). Nevertheless, the frequency of most bacterial phyla, except *Firmicutes* and *Actinobacteria*, was still significantly correlated with 2-weeks prior precipitation in the reduced precipitation treatment. However, the frequency of bacterial potential cellulose degraders was not correlated with precipitation in this treatment. In contrast, the frequency of sequences for fungi significantly increased under reduced precipitation (**Table 1**).

**FIGURE 3:**
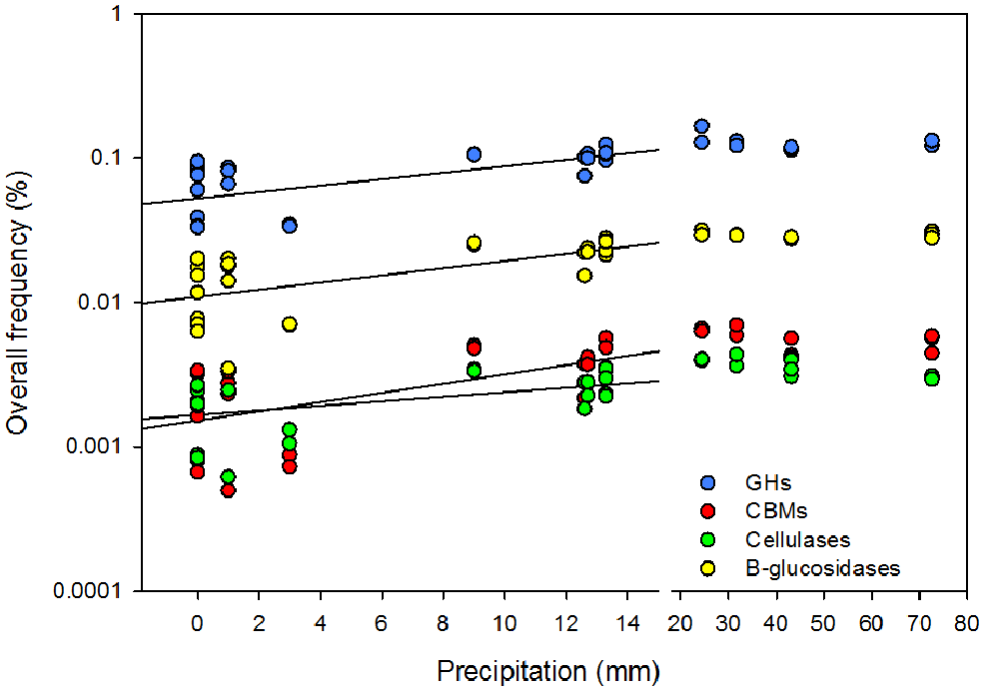
**Effect of the cumulative precipitation during the 2 weeks prior to litter collection on the overall rarefied frequency of microbial glycoside hydrolases (GHs), carbohydrates binding modules (CBMs), β-glucosidases, and cellulases.** Break in the *x*-axis was introduced to discriminate low from high precipitation.

Cellulase frequencies followed the responses of the bacterial community (**Figure 1C**). GHs (*r*_Pearson_ = 0.67), CBMs (*r*_Pearson_ = 0.66), β-glucosidases (*r*_Pearson_ = 0.67), and cellulases (*r*_Pearson_ = 0.49) were significantly correlated with cumulative precipitation (*P*_Pearson_ < 0.05), under low levels precipitation. Above ∼20 mm precipitation, trait frequency was unaffected by further increase in precipitation (**Figure 3**). We also analyzed how nitrogen deposition affected microbial community structure and the functional potential for litter deconstruction. Under increased nitrogen availability, the frequency of reads from most of the microbial lineages, except *Archaea*, and functional traits remained unchanged (**Table 1**; Table S3).

### LINKING MICROBIAL DIVERSITY TO FUNCTION

**FIGURE 4:**
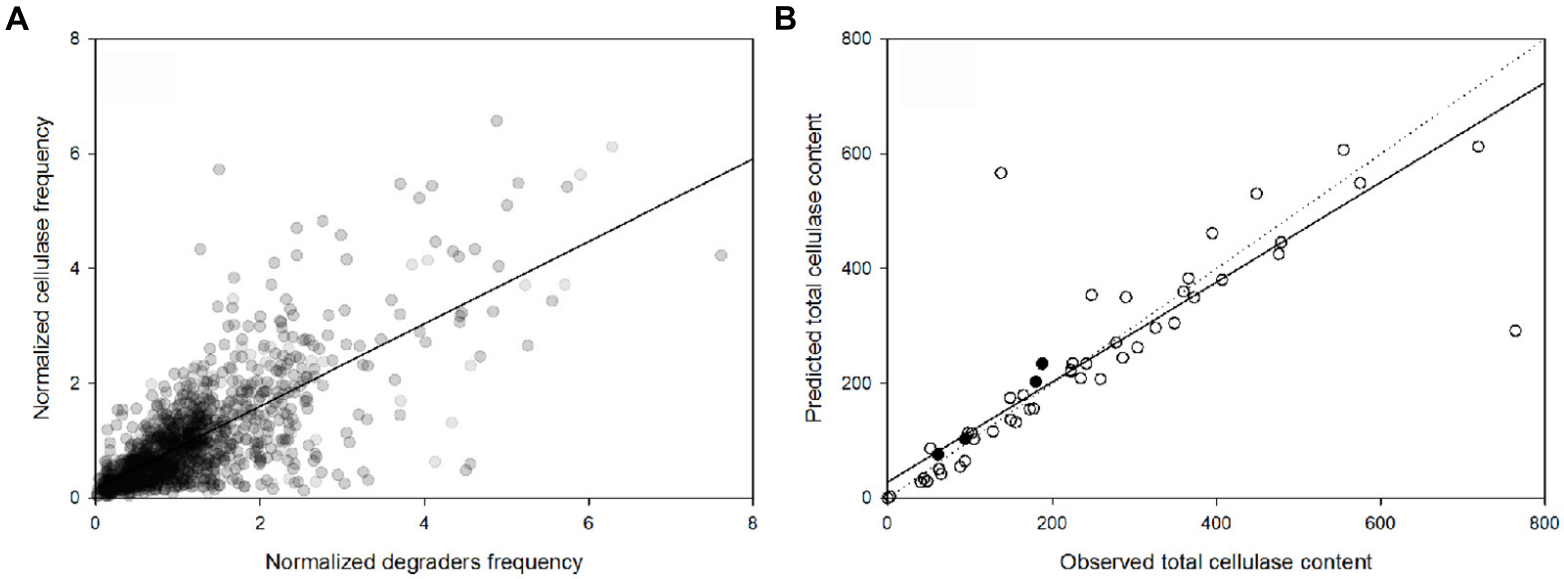
**(A)** Linear dependence of the normalized abundance of potential cellulose degraders and their potential cellulase content, across the samples (PSpearman < 0.01). **(B)** Comparison between the observed and the predicted cellulase content based on microbial community composition [samples used to construct the linear regression model (○) and independent samples from the factorial reduced precipitation and added nitrogen treatment (●)]. The solid and dotted lines are the regression line and the 1:1 line, respectively.

The genus-specific cellulase frequency was highly correlated with the abundance of cellulose-degrading genera (*P*_Spearman_ < 0.01; **Figure 4A**). Based on this relationship and the lineage-specific average frequency of cellulases (**Figure 2A**), the total cellulase content in each metagenome sample was highly predictable based on the microbial community composition (at the genus level) of the sample (*P*_Spearman_ < 0.01; **Figure 4B**). As an additional test, we predicted the total cellulase gene content in four samples derived from a combined nitrogen addition and reduced precipitation treatment that were not part of any of the previous analyses (**Figure 4B**). In these four samples, the predicted differed from the observed cellulase abundance by 14% (616 cellulases sequences predicted but 530 detected), in total.

In order to test for a relationship between drought or nitrogen responses and potential for cellulose deconstruction, we analyzed the correlation between rarefied reads for potential cellulase and log response ratios for abundance response to experimental precipitation reduction and increased nitrogen availability for the entire microbial community (at the genus level). In contrast to our hypothesis (i.e., correlation between cellulolytic traits and responses to precipitation and nitrogen availability), the frequency of cellulolytic traits were not correlated with response ratios (*P*_Spearman_ > 0.05).

## DISCUSSION

### PATTERNS IN DIVERSITY AND CELLULOLYTIC POTENTIAL

Generally, Fungi are assumed to be the most active microorganisms in litter deconstruction (Boer et al., 2005; Deacon et al., 2006; van der Wal et al., 2013). For example, a recent proteomic analysis suggests that Fungi express many hydrolytic enzymes (Schneider et al., 2012), and another study shows the incorporation of labeled substrates into mainly fungal biomarkers (van der Wal et al., 2013). Here, we observe that the majority of sequences for GHs in grassland leaf litter metagenomes are associated with bacterial lineages. This result is consistent with relatively high bacterial biomass in the leaf litter from Loma Ridge (Alster et al., 2013) and the representation of bacterial GHs in fosmid libraries derived from the same litter and shown to be active on plant polymers (Nyyssönen et al., 2013). As described for forest litter (Stursová et al., 2012), the microbial communities involved in cellulose deconstruction in grassland leaf litter may also be enriched in bacteria due to differences in leaf litter chemistry and climatic conditions. Thus, cellulolytic bacteria may be important for litter decomposition.

We detected sequences in leaf litter communities affiliated with every known cellulolytic GH family. In contrast, cellulases are less diverse in microbial communities involved in plant cell wall deconstruction in the cow rumen (Hess et al., 2011), a fungus garden (Suen et al., 2010), or the termite hindgut (Warnecke et al., 2007). Leaf litter potentially contains more complex and heterogeneous substrates and displays more fluctuations in environmental parameters (e.g., substrate chemistry and availability, temperature, and water/oxygen availability). This variation may lead to genetically and functionally diverse communities.

Many of the bacterial potential cellulose degraders we identified are commonly involved in cellulose deconstruction including Streptomyces (Semêdo et al., 2000), *Sorangium* (Hou et al., 2006), *Myxococcus* (Bensmail et al., 1998), *Acidothermus* (Barabote et al., 2009), and *Thermobispora* (Anderson et al., 2012). In most cases, these lineages are associated with cellulolytic activity resulting from the expression of multiple and diverse genes for cellulases (Wilson, 2011). In agreement with the CAZY-genome database (Lombard et al., 2014), some less frequent potential cellulose degraders harbor a higher frequency of reads for putative cellulases including *Herpetosiphon aurantiacus*, *Saccharophagus degradans*, and *Sorangium cellulosum*. This suggests that potential cellulolytic lineages that are less frequent may have an impact on litter deconstruction as shown for some specific fungal taxa (Deacon et al., 2006). On the other hand, the most abundant potential cellulose degraders (e.g., *Streptomyces*) display a lower frequency of cellulase sequences. This result is in good agreement with the hypothesis that reducing the number of cellulase genes in bacteria may reduce the cost of enzyme production and allow a higher growth rate (Allison, 2012). We speculate that such a high variability of the cellulolytic potential, together with other adaptations [i.e., filamentous growth of *Streptomyces* (Boer et al., 2005) and cellulolosome production of *Saccharophagus* and *Clostridium* (Sabathe et al., 2002; Taylor et al., 2006)] increases the decomposition of plant material by providing synergistic biochemical activities that target different fractions of the substrate (Hättenschwiler et al., 2011; van der Wal et al., 2013).

Sequences for putative β-glucosidases in bacterial potential cellulose degraders account for 52% of the detected sequences from GH families 1 and 3. As previously suggested, it is likely that these enzymes are broadly distributed in bacteria including many non-cellulolytic lineages (i.e., opportunists or cheaters; Berlemont and Martiny, 2013). Indeed, many bacterial lineages, including some non-degraders, are stimulated when subjected to labile oligosaccharides (Goldfarb et al., 2011). As a consequence, β-glucosidase activity in environmental samples is likely a poor proxy for the degradation of complex cellulose polymers. However, opportunists may contribute indirectly to plant litter degradation by processing cellulose deconstruction byproducts.

### MICROBIAL RESPONSE TO ENVIRONMENTAL MANIPULATIONS

Our data are consistent with prior findings suggesting that Fungi are less negatively affected by low water availability than bacterial populations and are thus more frequent under seasonally occurring or experimental drought (Allison et al., 2013; Barnard et al., 2013; van der Wal et al., 2013). Indeed, experimental precipitation reduction marginally, but significantly, increases fungal relative abundance. At our site, this increased frequency of reads from fungi under dry conditions is likely to result from a reduction in bacterial biomass (Allison et al., 2013). Although most bacterial relative abundances declined under low water availability, there was some variation in response at the genus level within the *Actinobacteria* and *Firmicutes*. In some genera within these phyla, tolerance to desiccation is likely achieved through multiple strategies to survive reduced water potential [e.g., production of a surfactant in *Bacillus* (Straight et al., 2006), exopolysaccharides in *Pseudomonas* (Roberson and Firestone, 1992), osmolytes in *Cyanobacteria* (Rajeev et al., 2013)].

### LINKS BETWEEN TAXONOMY AND FUNCTION

Across seasons and treatments, bacterial responses to reduced precipitation and nitrogen addition were not directly correlated with cellulolytic potential. However, the ratio of Bacteria to Fungi was reduced under seasonal drought and experimental precipitation reduction. Fungi have relatively few cellulases, and litter decomposition experiments show that litter decay rates are lower during seasonal drought periods and in the reduced precipitation treatment (Allison et al., 2013). This pattern could be interpreted as a reduced role of Fungi in comparison to Bacteria in litter decomposition in this ecosystem, but further studies are needed to quantify the contribution from the two groups. Also contrary to our physiological tradeoffs hypothesis, copiotrophic Bacteria that were favored in nitrogen-enriched plots did not show higher genetic cellulolytic potential.

Consistent with our initial hypothesis, the genetic potential for cellulose deconstruction was highly predictable based on the taxonomic composition of the microbial community. This finding aligns with the previously described conservatism at the genus-species level of genes for cellulases in sequenced bacterial genomes (Berlemont and Martiny, 2013). Our current study generalizes this pattern to diverse communities containing poorly sequenced (e.g., *Acidobacteria* and *Chloroflexi*) or hyper-variable taxa (e.g., *Bacillus*; Berlemont and Martiny, 2013), thereby reducing the dependence of trait prediction on sequenced genomes. Furthermore, a phylogeny-function relationship allows for the prediction of the cellulolytic potential in taxonomically resolved communities.

The sequencing of metagenomes involves some important potential limitations. The approach may have a bias due to variations in extraction efficiency among different lineages. Also, poorly characterized bacterial phyla (e.g., *Acidobacteria*) and complex genomes from fungi are unevenly detected in metagenomic studies due the reliance on a small number of previously annotated genes and genomes. Thus, the frequencies and metabolic potentials of phyla with few genome sequences are likely underestimated. In addition, we specifically recognize that some genes from GHs identified as potential cellulases may possibly have other enzymatic functions (Berlemont and Martiny, 2013; Nyyssönen et al., 2013). In addition, some enzymes identified as cellulases may be involved in cellulose biosynthesis or in the interaction between microorganisms and plants (Medie et al., 2012; Berlemont and Martiny, 2013). For example, genera described as plant pathogens [e.g., *Clavibacter* (Jahr et al., 2000)] or plant growth promoting rhizo-bacteria [e.g., *Rhizobium* (Robledo et al., 2008)] may not contribute to cellulose degradation. As is the case for most metagenomic analyses, these biases may influence our results in unknown ways as most traits have not been fully characterized genetically and/or biochemically. Despite these caveats, our metagenomic approach provides a powerful tool for linking microbial community composition and potential function under environmental change. When combined with experimental confirmation of biochemical function (Nyyssönen et al., 2013), highly robust linkages between microbial composition and ecosystem processes may be achieved.

Our data show that fungi are drought resistant, but they are likely not the primary contributors to the cellulolytic potential in this grassland litter community. Rather, changes in cellulolytic potential due to seasonality and experimental precipitation reduction are driven by the dynamics of bacterial taxa that are highly sensitive to drought. Together with previous study (Allison et al., 2013), these results suggest that drought-associated reductions in litter decay and cellulose deconstruction may be related to shifts in microbial community composition and not simply direct moisture limitation. Under nitrogen addition, litter decay is not likely to increase through effects on cellulolytic potential. Describing the effect of precipitation reduction and nitrogen deposition, across seasons, on microbial communities involved in plant material deconstruction is a prerequisite for future investigation of combined effects of these perturbations. More broadly, the phylogenetic conservatism of functional traits and the response of microbial taxa to simulated environmental changes provide a robust conceptual framework to predict how microbial communities will respond to global changes and impact ecosystem functioning.

## Conflict of Interest Statement

The authors declare that the research was conducted in the absence of any commercial or financial relationships that could be construed as a potential conflict of interest.
