# Supplementary material for: Cellulolytic potential under environmental changes in microbial communities from grassland litter
Source: Front Microbiol. 2014 Nov 25;5:639. doi: 10.3389/fmicb.2014.00639 (PMC4243572; doi:10.3389/fmicb.2014.00639)
Supplement: Supplementary file 1 [file Data_Sheet_1.DOC]

# Cellulolytic potential under environmental changes in microbial communities from grassland litter

1. Renaud Berlemont^1^, Steven D. Allison^1,2^, Claudia Weihe^2^, Ying Lu^2^, Eoin L. Brodie^3,4^, Jennifer B.H. Martiny^2^, & Adam C. Martiny^1,2*^
2. ^1^Department of Earth System Science and ^2^Department of Ecology and Evolutionary Biology, University of California, Irvine, California, USA. ^3^Ecology Department, Earth Sciences Division, Lawrence Berkeley National Laboratory, Berkeley, California, USA. ^4^Department of Environmental Science, Policy and Management, University of California, Berkeley, California, USA.
3. * Correspondence: A.C. Martiny, Department of Earth System Science, 3208 Croul Hall, University of California, 92697 Irvine, California, USA.
4. E-mail: amartiny@uci.edu

Supporting Material.

Figure S1. Distribution of reads for (A) glycoside hydrolases (GHs) and (B) carbohydrate binding modules (CBMs). (C) Identity of the detected sequences for GHs/CBMs (n=460,379), cellulases (n=12,680), and β-glucosidases (n=104,088) to their best hit in the Pfam-database.


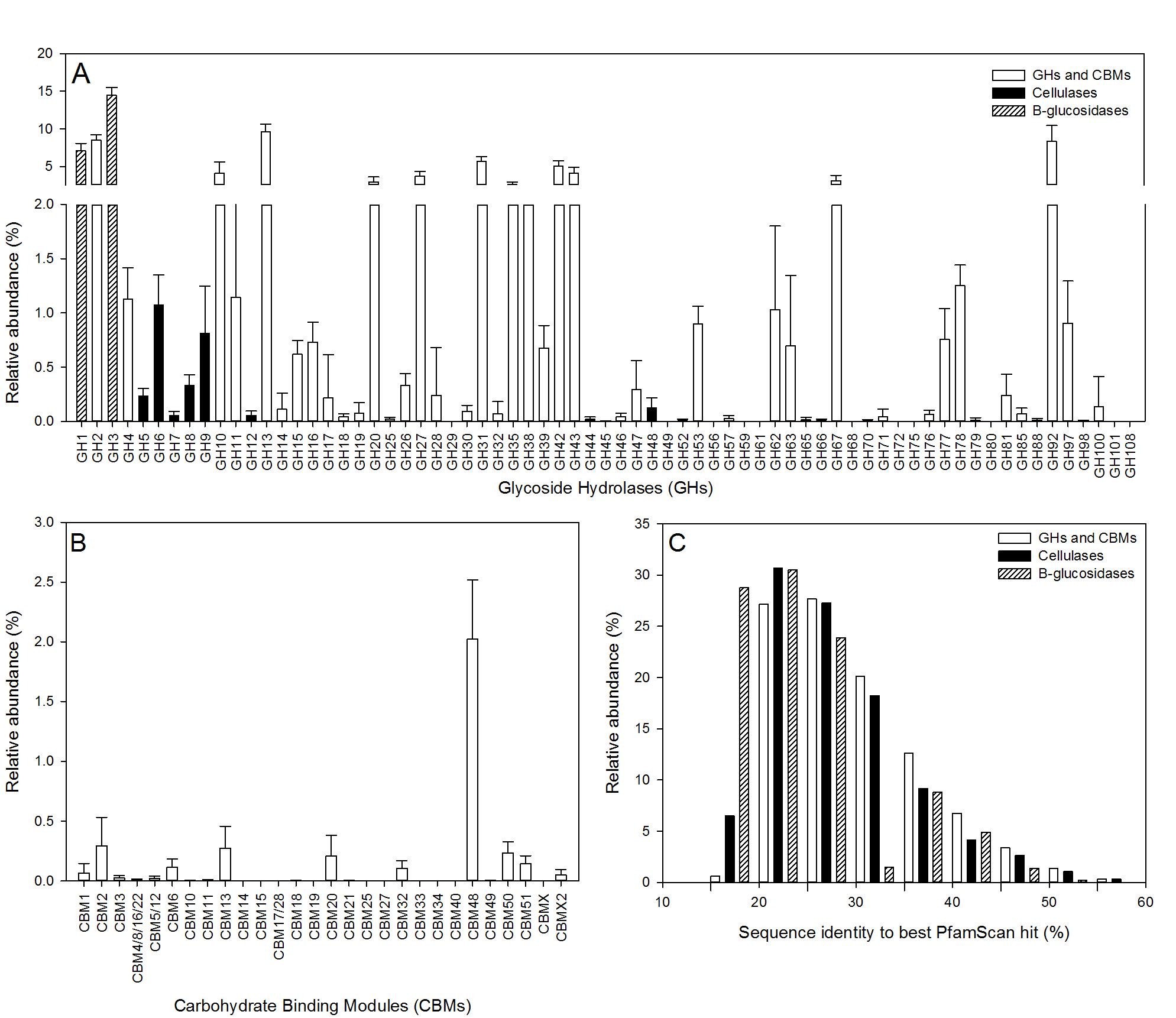


Figure S2. Sequencing effort of the leaf litter metagenomes. Hashed areas represent sequences that passed the quality control. The sampling dates are Ap10 (14-Apr-10), Au10 (20-Aug-10), D10 (17-Dec-10), F11 (28-Feb-11), J11 (10-jun-10), S11 (21-Sep-11), D11 (14-Dec-11), and M12 (12-Mar-12). (*) samples not considered for statistical analysis.


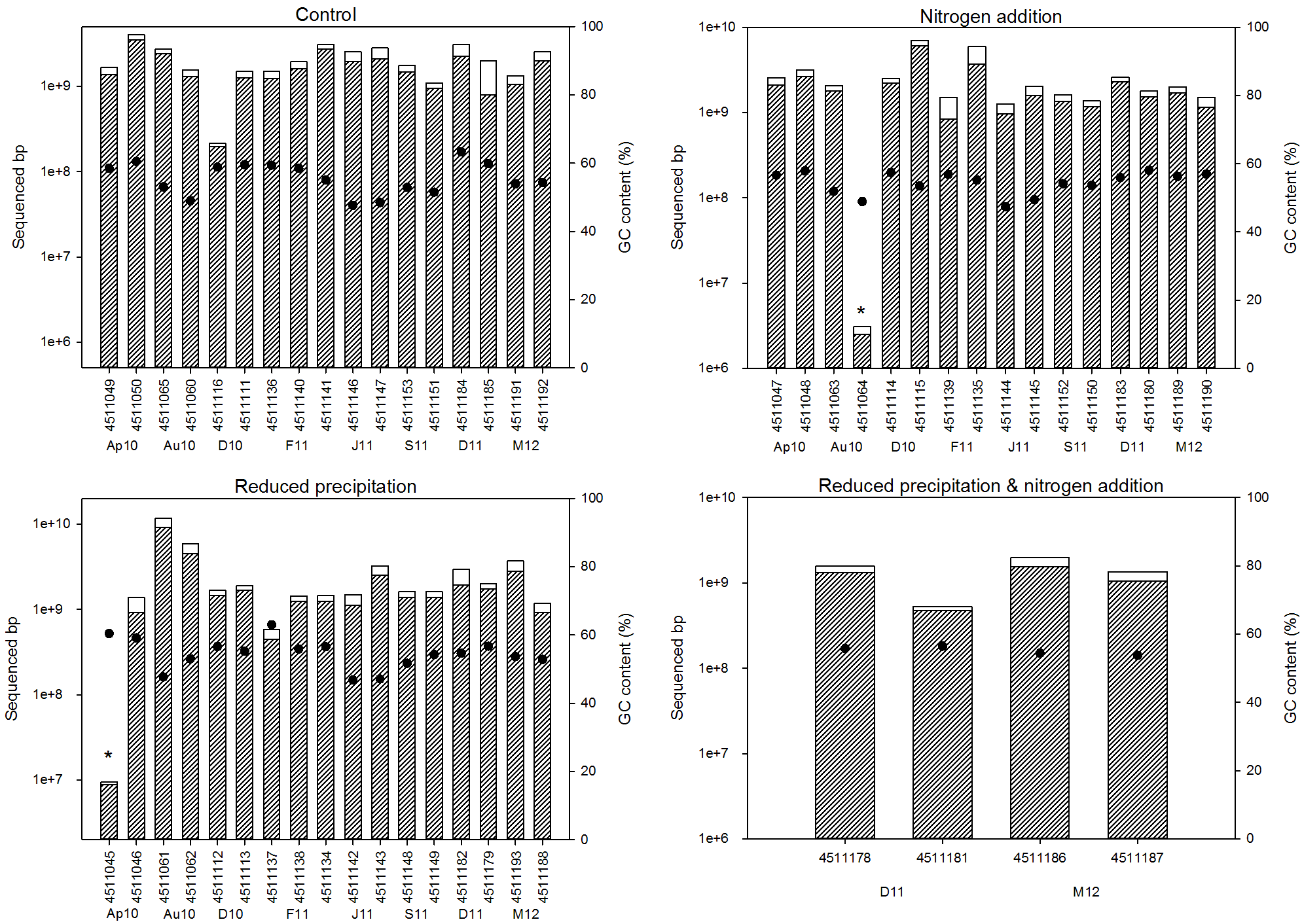


Table S1. Abundance profile of sequences for Glycoside Hydrolases from the leaf litter metagenome. Asterisks denote GH families with known cellulolytic activity.

| GH family | Pfam Model | Sequence Count |  | GH family | Pfam Model | Sequence Count |
| --- | --- | --- | --- | --- | --- | --- |
| GH1 | PF00232 | 41075 |  | GH46 | PF01374 | 378 |
| GH2 | PF00703 PF02836 PF02837 | 46439 |  | GH47 | PF01532 | 1243 |
| GH3 | PF00933 PF01915 | 80000 |  | GH48* | PF02011 | 1038 |
| GH4 | PF02056 PF11975 | 7295 |  | GH49 | PF03718 | 0 |
| GH5* | PF00150 | 1334 |  | GH52 | PF03512 | 60 |
| GH6* | PF01341 | 5501 |  | GH53 | PF07745 | 4805 |
| GH7* | PF00840 | 213 |  | GH56 | PF01630 | 0 |
| GH8* | PF01270 | 1888 |  | GH57 | PF03065 | 368 |
| GH9* | PF00759 | 4267 |  | GH59 | PF02057 | 0 |
| GH10 | PF00331 | 20211 |  | GH61 | PF03443 | 0 |
| GH11 | PF00457 | 4390 |  | GH62 | PF03664 | 4380 |
| GH12* | PF01670 | 360 |  | GH63 | PF03200 | 2488 |
| GH13 | PF00128 | 56579 |  | GH65 | PF03633 PF03632 PF03636 | 171 |
| GH14 | PF01373 | 444 |  | GH66 | PF13199 | 74 |
| GH15 | PF00723 | 4028 |  | GH67 | PF07477 PF07488 PF03648 | 17675 |
| GH16 | PF00722 | 3922 |  | GH68 | PF02435 | 0 |
| GH17 | PF00332 | 663 |  | GH70 | PF02324 | 45 |
| GH18 | PF00704 | 243 |  | GH71 | PF03659 | 149 |
| GH19 | PF00182 | 325 |  | GH72 | PF03198 | 1 |
| GH20 | PF00728 PF02838 | 16701 |  | GH75 | PF07335 | 0 |
| GH25 | PF01183 | 109 |  | GH76 | PF03663 | 354 |
| GH26 | PF02156 | 1943 |  | GH77 | PF02446 | 5378 |
| GH27 | PF02065 | 19030 |  | GH78 | PF05592 | 6951 |
| GH28 | PF00295 | 1099 |  | GH79 | PF03662 | 28 |
| GH29 | PF01120 | 0 |  | GH80 | PF13647 | 0 |
| GH30 | PF02055 | 550 |  | GH81 | PF03639 | 965 |
| GH31 | PF01055 | 30280 |  | GH85 | PF03644 | 289 |
| GH32 | PF08244 PF00251 | 432 |  | GH88 | PF07470 | 69 |
| GH35 | PF01301 | 13987 |  | GH92 | PF07971 | 46177 |
| GH38 | PF01074 PF07748 | 12980 |  | GH97 | PF10566 | 5384 |
| GH39 | PF01229 | 3841 |  | GH98 | PF08307 PF08306 | 28 |
| GH42 | PF02449 PF08533 PF08532 | 27752 |  | GH100 | PF12899 | 401 |
| GH43 | PF04616 | 20819 |  | GH101 | PF12905 | 0 |
| GH44* | PF12891 | 203 |  | GH108 | PF05838 | 0 |
| GH45* | PF02015 | 17 |  | GHcc | PF11790 | 31 |

Table S2. Abundance profile of sequences for Carbohydrate Binding Modules from the leaf litter metagenome

| CBM family | Pfam Model | Sequence Count |  | CBM family | Pfam Model | Sequence Count |
| --- | --- | --- | --- | --- | --- | --- |
| CBM1 | PF00734 | 227 |  | CBM20 | PF00686 | 814 |
| CBM2 | PF00553 | 2288 |  | CBM21 | PF03370 | 5 |
| CBM3 | PF00942 | 163 |  | CBM25 | PF03423 | 4 |
| CBM4 - 8 - 16 - 22 | PF02018 | 17 |  | CBM27 | PF09212 | 0 |
| CBM5/12 | PF02839 | 121 |  | CBM32 | PF00754 | 699 |
| CBM6 | PF03422 | 738 |  | CBM33 | PF03067 | 0 |
| CBM10 | PF02013 | 15 |  | CBM34 | PF02903 | 2 |
| CBM11 | PF03425 | 11 |  | CBM40 | PF02973 | 1 |
| CBM13 | PF00652 | 2223 |  | CBM48 | PF02922 | 12464 |
| CBM14 | PF01607 | 0 |  | CBM49 | PF09478 | 9 |
| CBM15 | PF03426 | 3 |  | CBM50 | PF01476 | 1503 |
| CBM17/28 | PF03424 | 0 |  | CBM51 | PF08305 | 964 |
| CBM18 | PF00187 | 1 |  | CBMX | PF06204 | 0 |
| CBM19 | PF03427 | 0 |  | CBMX2 | PF03442 | 186 |

Table S3. Average frequency (%) of rarefied reads for the most abundant bacterial genus of potential cellulose degraders in leaf litter metagenomes. Significant treatment effects relative to controls are denoted with an asterisk (**P*<0.05, paired Welch *t*-test). Effect of cumulative precipitation (Precip., r_Pearson_) on the frequency of lineages in each treatment (**P*_Pearson_<0.05).

|  | Control | | Reduced precipitation | | Nitrogen addition | |
| --- | --- | --- | --- | --- | --- | --- |
|  | Freq. (%) | Precip.r_P_ (**P*_P_) | Freq. (%)  (^*^*P*_Welch-_*_t_*) | Precip. r_P_ (**P*_P_) | Freq. (%) (^*^*P*_Welch-_*_t_*) | Precip. r_P_ (**P*_P_) |
| **Sum of Bacteria** | **61.46 ±22.6** | **0.67*** | **50.38 ±20.58**^+^ | **0.76*** | **63.63 ±18.14** | **0.68*** |
| *Proteobacteria* | 27.96 ±11.61 | 0.75* | 21.81 ±9.96^+^ | 0.81* | 29.15 ±10.46 | 0.74* |
| *Methylobacterium* | 1.61 ±0.84 | 0.49* | 1.04 ±0.5 | 0.84* | 1.4 ±0.52 | 0.59* |
| *Burkholderia* | 1.32 ±0.47 | 0.77* | 1.1 ±0.38 | 0.83* | 1.26 ±0.41 | 0.75* |
| *Rhizobium* | 0.99 ±0.56 | 0.72* | 0.83 ±0.52 | 0.87* | 1.02 ±0.54 | 0.53* |
| *Caulobacter* | 0.66 ±0.34 | 0.72* | 0.5 ±0.25 | 0.79* | 0.68 ±0.32 | 0.66* |
| *Xanthomonas* | 0.54 ±0.24 | 0.71* | 0.43 ±0.19 | 0.8* | 0.57 ±0.28 | 0.87* |
| *Sorangium* | 0.19 ±0.09 | 0.72* | 0.12 ±0.05 | 0.71* | 0.16 ±0.07 | 0.48* |
| *Myxococcus* | 0.14 ±0.06 | 0.53* | 0.11 ±0.04 | 0.63* | 0.13 ±0.05 | 0.53* |
| *Cellvibrio* | 0.05 ±0.02 | 0.79* | 0.05 ±0.02 | 0.7* | 0.05 ±0.03 | 0.68* |
| *Xylella* | 0.05 ±0.02 | 0.76* | 0.04 ±0.02 | 0.74* | 0.05 ±0.02 | 0.75* |
| *Saccharophagus* | 0.05 ±0.02 | 0.74* | 0.04 ±0.01 | 0.74* | 0.05 ±0.02 | 0.69* |
| *Actinobacteria* | 22.46 ±9.67 | 0.32* | 19.5 ±9.28 |  | 22.85 ±6.41 |  |
| *Streptomyces* | 2.86 ±1.11 | 0.32* | 2.53 ±0.95 |  | 2.61 ±0.66 |  |
| *Clavibacter* | 2.69 ±1.12 |  | 2.56 ±1.17 |  | 3.36 ±1.54 |  |
| *Mycobacterium* | 1.65 ±0.72 | 0.43* | 1.29 ±0.55 | 0.76* | 1.51 ±0.47 | 0.4* |
| *Nocardioides* | 1.04 ±0.27 |  | 1.12 ±0.43 |  | 1.03 ±0.08 |  |
| *Kineococcus* | 0.74 ±0.48 |  | 0.85 ±0.4 |  | 0.66 ±0.36 |  |
| *Salinispora* | 0.43 ±0.26 | 0.44* | 0.34 ±0.23 |  | 0.38 ±0.17 |  |
| *Cellulomonas* | 0.28 ±0.13 | 0.2* | 0.27 ±0.12 |  | 0.29 ±0.09 |  |
| *Actinosynnema* | 0.26 ±0.12 | 0.27* | 0.23 ±0.1 |  | 0.25 ±0.07 | 0.23* |
| *Streptosporangium* | 0.24 ±0.11 | 0.37* | 0.2 ±0.1 | 0.63* | 0.22 ±0.07 |  |
| *Catenulispora* | 0.23 ±0.1 | 0.4* | 0.2 ±0.08 |  | 0.21 ±0.06 |  |
| *Xylanimonas* | 0.21 ±0.09 | 0.23* | 0.2 ±0.08 |  | 0.21 ±0.07 |  |
| *Nocardiopsis* | 0.16 ±0.07 | 0.31* | 0.15 ±0.06 | 0.71* | 0.16 ±0.05 |  |
| *Thermobifida* | 0.14 ±0.06 | 0.4* | 0.13 ±0.06 | 0.67* | 0.13 ±0.05 | 0.33* |
| *Acidothermus* | 0.12 ±0.06 | 0.35* | 0.11 ±0.05 | 0.64* | 0.12 ±0.04 |  |
| *Jonesia* | 0.12 ±0.05 | 0.31* | 0.11 ±0.04 |  | 0.12 ±0.04 |  |
| *Thermobispora* | 0.1 ±0.04 | 0.36* | 0.09 ±0.04 |  | 0.09 ±0.03 |  |
| *Bacteroidetes* | 5.29 ±3.15 | 0.89* | 4 ±2.31 | 0.72* | 6.15 ±3.38 | 0.81* |
| *Spirosoma* | 0.76 ±0.57 | 0.87* | 0.36 ±0.19 | 0.66* | 0.74 ±0.42 | 0.86* |
| *Flavobacterium* | 0.43 ±0.3 | 0.88* | 0.35 ±0.26 | 0.75* | 0.52 ±0.38 | 0.85* |
| *Dyadobacter* | 0.4 ±0.23 | 0.76* | 0.35 ±0.22 | 0.69* | 0.53 ±0.31 | 0.59* |
| *Cytophaga* | 0.32 ±0.21 | 0.86* | 0.25 ±0.14 | 0.67* | 0.36 ±0.23 | 0.74* |
| *Firmicutes* | 2.5 ±0.19 |  | 2.4 ±0.48 |  | 2.43 ±0.25 |  |
| *Bacillus* | 0.42 ±0.03 |  | 0.38 ±0.04 |  | 0.42 ±0.05 |  |
| *Clostridium* | 0.3 ±0.03 |  | 0.3 ±0.09 |  | 0.29 ±0.03 |  |
| *Chloroflexi* | 0.36 ±0.09 | 0.55* | 0.3 ±0.06^+^ | 0.78* | 0.33 ±0.06 | 0.51* |
| *Herpetosiphon* | 0.05 ±0.01 | 0.45* | 0.04 ±0.01 | 0.64* | 0.04 ±0.01 | 0.44* |

Table S4. Significance of treatments (reduced precipitation or nitrogen addition), sampling dates, and interaction on the distribution of reads with best hit in taxonomically (phylum) or functionally (potential cellulose degraders) defined groups in the leaf litter metagenomes. Linear mixed effects models were analyzed using ANOVA (significance level: **P*<0.05 and ^+^*P*<0.1).

|  | **Treatment** | | **Date** | | **Treatment X Date** | |
| --- | --- | --- | --- | --- | --- | --- |
|  | ***P*** | ***F*** | ***P*** | ***F*** | ***P*** | ***F*** |
| **Sum of Bacteria** |  |  | * | 42.3640 |  |  |
| *Proteobacteria* | + | 5.70 | * | 95.38 | + | 2.1161 |
| *Actinobacteria* |  |  | * | 13.05 |  |  |
| *Bacteroidetes* |  |  | * | 48.50 | * | 2.20 |
| *Firmicutes* |  |  | * | 2.82 |  |  |
| *Cyanobacteria* | + | 5.37 | * | 20.04 |  |  |
| *Chloroflexi* |  |  | * | 12.84 |  |  |
| *Acidobacteria* |  |  | * | 49.97 |  |  |
| *Planctomycetes* | + | 6.69 | * | 23.17 | + | 2.10 |
| *Verrucomicrobia* | + | 5.23 | * | 31.77 |  |  |
|  | | | | | | |
| **Sum of Pot. Cell. Degraders** | + | 5.74 | * | 22.81 |  |  |
|  | | | | | | |
| **Sum of Archaea** | + | **6.50** | ***** | **10.18** |  |  |
| *Euryarchaeota* | + | 7.54 | * | 5.48 |  |  |
| *Crenarchaeota* |  |  | * | 24.37 |  |  |
| *Thaumarchaeota* |  |  | * | 12.61 |  |  |
|  | | | | | | |
| **Sum of Fungi** | + | **7.79** | ***** | **19.25** |  |  |
| *Ascomycota* | + | 8.17 | * | 18.66 |  |  |
| *Basidiomycota* |  |  | * | 12.08 |  |  |
| *Unclassified Fungi* |  |  | * | 8.53 |  |  |
| *Glomeromycota* |  |  | * | 4.65 |  |  |

Table S5. MG-RAST accession numbers for the leaf litter metagenome. (^+^) denote samples that were sequenced twice in order to assess the consistency between the sequencing runs. (^++^) samples used to estimate the global cellulolytic potential according to the taxonomy, only.

| Treatment | Control | Reduced Precipitations | Nitrogen Addition | Nitrogen Addition / Reduced Precipitations |
| --- | --- | --- | --- | --- |
| April 2010 (Ap10) | 4511049  4511050 | 4511045  4511046 | 4511047  4511048 |  |
| August 2010 (Au10) | 4511065  4511060 | 4511061  4511062 | 4511063  4511064 |
| December 2010  (D10) | 4511116  4511111 | 4511112  4511113 | 4511114  4511115 |
| February 2011  (F11) | 4511136  4511140  4511141^+^ | 4511137  4511138  4511134^+^ | 4511139  4511135 |
| June 2011  (J11) | 4511146  4511147 | 4511142  4511143 | 4511144  4511145 |
| September 2011  (S11) | 4511153  4511151 | 4511148  4511149 | 4511152  4511150 |
| December 2011  (D11) | 4511184  4511185 | 4511182  4511179 | 4511183  4511180 | 4511181^++^  4511178^++^ |
| March 2012  (M12) | 4511191  4511192 | 4511193  4511188 | 4511189  4511190 | 4511186^++^  4511187^++^ |
